# Anaplastic Lymphoma Kinase-Tyrosine Kinase Inhibitor Treatment for Metastatic Non-small Cell Lung Cancer Throughout the Entire Gestation Period

**DOI:** 10.7759/cureus.63592

**Published:** 2024-07-01

**Authors:** Mari Kato, Takumi Yamaura, Hayato Mine, Shogo Kin, Hiroyuki Suzuki

**Affiliations:** 1 Obstetrics and Gynecology, Takeda General Hospital, Fukushima, JPN; 2 Thoracic Surgery, Takeda General Hospital, Fukushima, JPN; 3 Thoracic Surgery, Fukushima Medical University, Fukushima, JPN; 4 Obstetrics and Gynecology, Takeda General Hospital, Aizuwakamatsu, JPN

**Keywords:** tyrosine kinase inhibitor, anaplastic lymphoma kinase, case report, non-small cell lung cancer, pregnancy, alectinib, alk

## Abstract

Non-small cell lung cancer (NSCLC) occasionally develops in younger, fertile patients. This early-onset NSCLC tends to have more oncogenic driver mutations than in aged patients. Among early-onset NSCLC patients, pregnancy is very rare. However, there are some patients who were able to balance tyrosine kinase inhibitor (TKI) administration and pregnancy. Here, we report a case of a pregnancy under alectinib hydrochloride (a second-generation anaplastic lymphoma kinase (ALK)-TKI) administration throughout the entire gestational period for ALK*-*rearranged metastatic lung adenocarcinoma. The patient was an Asian female in her early 20s who became aware of her pregnancy after diagnosis and the start of alectinib administration. She intended to have the baby despite the necessity of continuing her treatment and the unknown risks involved. A multidisciplinary team (thoracic surgeon, obstetrics, pediatrics, and so on) was organized to support the patient, baby, and family. There were no obvious signs of tumor progression during pregnancy. She gave birth at 41 weeks and one day of gestation. There was no placental metastasis. Alectinib concentration at delivery was 155 ng/mL in maternal blood, 22.1 ng/mL in umbilical cord venous blood, 20.1 ng/mL in amniotic fluid, and 11.8 ng/mL in colostrum. The baby had been exposed to alectinib throughout the entire pregnancy; however, fetal growth curve parameters remained within the normal ranges and the baby developed without anatomical or neurodevelopmental anomalies or fetal metastasis for the first 13 months of age.

## Introduction

Non-small cell lung cancer (NSCLC) occasionally develops in younger patients, and this early-onset NSCLC has been associated with factors such as no smoking history, female sex, advanced cancer stage, and a high frequency of targetable genomic alterations [1]. There are only a few reports of cases of pregnant patients with metastatic NSCLC who received anticancer agents during the middle or late stage of pregnancy [2-5]. There are a few cases of patients who had received molecular targeted agents such as anaplastic lymphoma kinase (ALK)-tyrosine kinase inhibitor (TKI) throughout the entire pregnancy, including the organogenetic period. An important problem is that data are lacking regarding the safety ofALK-TKI administration for the fetus during the first trimester. Here, we report a case of a pregnant patient with ALK-rearranged metastatic NSCLC who was successfully treated with alectinib hydrochloride (alectinib) and delivered a baby that had been exposed to alectinib for the entire gestation period.

## Case presentation

**Figure 1:**
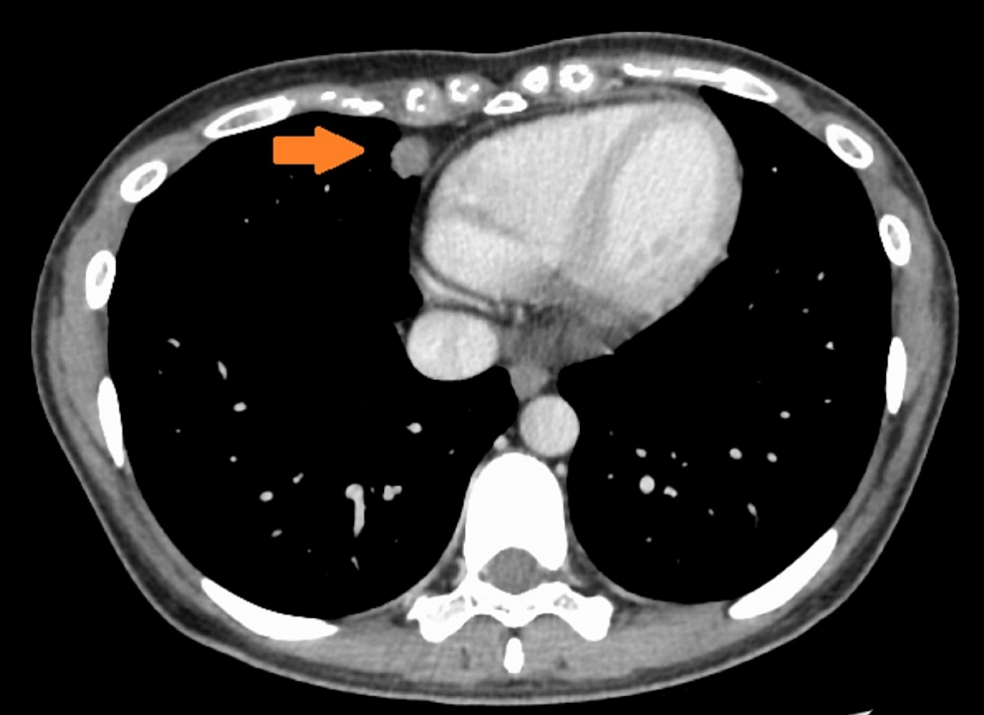
CT scan of the chest. The primary site at the initial examination.

**Figure 2:**
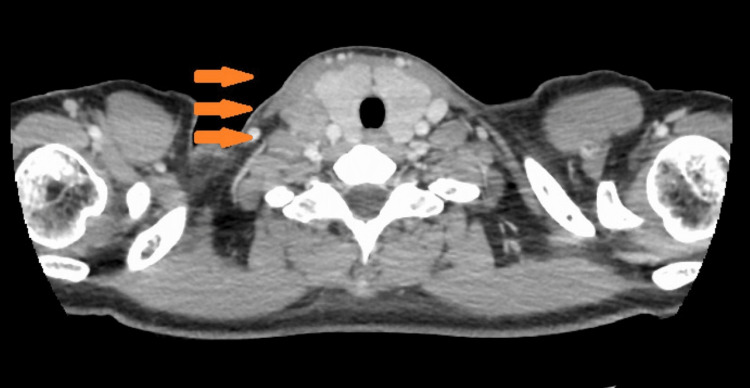
CT of the neck. Supraclavicular fossa lymph node at the initial examination.

**Figure 3:**
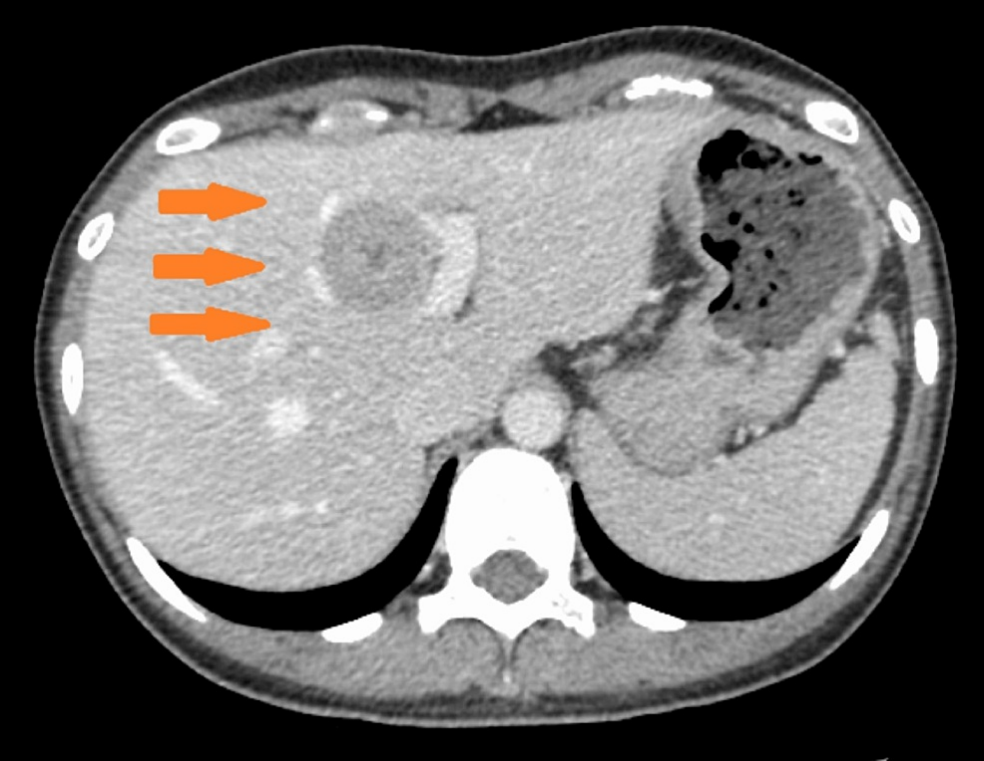
CT scan of the abdomen. Liver tumor at initial diagnosis.

A 22-year-old Asian female presented for consultation with the chief complaint of right cervical swelling. A fine needle biopsy suggested malignancy. She had never smoked and there was no history of juvenile-onset malignancy in her family. Computed tomography scans noted a 1.5 cm solid nodule in the middle lobe of the right lung (Figure 1), enlarged mediastinal and supraclavicular lymph nodes (Figure 2), and a liver nodule (Figure 3).

A right supraclavicular lymph node biopsy revealed a poorly differentiated adenocarcinoma, and she was diagnosed with c-T1bN3M1b (HEP), stage IVA. Driver oncogenic mutation examination showed ALK fusion in the tumor. Alectinib administration was started as the initial treatment after her oocytes were cryopreserved. Nine weeks later, she realized that she was seven weeks pregnant. The ethics committee and a multidisciplinary team, including oncologists, obstetricians, pediatricians, and a psychiatrist, met to discuss how to proceed. Since she was in the early stage of pregnancy, she believed that it would be unfeasible to delay treatment until delivery. She and her family wanted to continue the pregnancy despite the elevated risk of fetal malformation, abortion, and other unexpected events, and continued alectinib administration. She was successfully treated without any adverse events during the pregnancy, and the fetal growth parameters were also well within normal ranges for estimated fetal size. She delivered the baby spontaneously at 41 weeks and one day of gestation. The baby was 51 cm in height and weighed 2766 g, with no external deformity. There was no placental metastasis. The pathology diagnosis was umbilical corditis. Alectinib concentration at delivery was 155 ng/mL in maternal blood, 22.1 ng/mL in umbilical cord venous blood, 20.1 ng/mL in amniotic fluid, and 11.8 ng/mL in colostrum. As of 17 months after diagnosis, the mother has continued alectinib treatment. The baby has been developing without any problems in neurodevelopment or fetal metastasis as of 13 months of age.

## Discussion

Malignancy complicates about 1/1,000 pregnancies, and complications due to NSCLC are very rare. A total of 4% of all NSCLC cases develop in patients under the age of 40 years and about half have a targetable driver oncogenic mutation, mainly ALK(19%) and epidermal growth factor receptor (EGFR) (32%) [4]. Moreover, advanced maternal age could lead to an increased number of pregnancies in patients with ALK rearrangement in NSCLC.

An important problem is that ALK can be involved in fetal neurodevelopment [1]. However, in the previous three cases and the present case, there were no neurodevelopmental problems or malformations in 10 to 33-month-old children exposed to alectinib (Table 1).

**Table 1:** Ratio of umbilical cord blood concentration to maternal blood concentration of alectinib.

Author	Year	Age (years)	Umbilical cord (ng/mL)	Maternal blood (ng/mL)	Ratio
Present case	2022	Early 20s	22.1	155	14.3%
Scarfone et al. [2]	2021	31	18.0	259.0	6.9%
Shang et al. [3]	2023	35	69.2	299.0	23.1%
Boudy et al. [4]	2021	42	40	482	8.3%

In the present case, the ratio of exposure for the baby (umbilical cord blood to maternal blood alectinib concentration) was 14.3%. In other reported cases, the ratio was 6.94-23.14% (Table 2).

**Table 2:** Case series of patients with ALK rearrangement in NSCLC who had received alectinib throughout the entire pregnancy. ALK: anaplastic lymphoma kinase; NSCLC: non-small cell lung cancer; ADC: adenocarcinoma; SRT: stereotactic radiotherapy.

Author	Year	Age (years)	Subtype/metastases/stage	Treatment	Pregnancy outcomes	Malformation	Neural development problem	Maternal outcomes
Scarfone et al. [2]	2021	31	ADC/unknown/IV	Alectinib	C-section at 35 weeks	Lateral cervical fistula	None, 19 months	32 months after diagnosis. Still in partial response
Weidenbaum et al. [5]	2022	27	ADC/brain/IV	SRT for brain metastases, alectinib	(1) Vaginal at 39 weeks. (2) Vaginal at 39 weeks	N/A	None, (1) 27 months, (2) 10 months	3 months after the delivery of the second child, progression in the brain
Present case	2023	Early 20s	ADC/liver/IV	Alectinib	Vaginal at 41 weeks	N/A	None, 13 months	Ongoing treatment one year after delivery

Safety information on alectinib administration and fetal malformation during the first trimester of pregnancy is lacking. However, alectinib administration can be an option for patients with ALK rearrangement in NSCLC in the early phase of pregnancy.

## Conclusions

Pregnancy and childbirth during alectinib administration are very rare but possible. Since there are only a few known cases and many possible yet unknown risks, careful decision-making to determine whether to continue the pregnancy and how to continue treatment is necessary.
